# Relationships between internet addiction, smartphone addiction, sleep quality, and academic performance among high-school students

**DOI:** 10.1590/1806-9282.20230868

**Published:** 2024-03-04

**Authors:** Yasin Guclu, Ozge Aydin Guclu, Hakan Demirci

**Affiliations:** 1 Soğanlı Family Health Center, Department of Family Medicine – Bursa, Turkey.; 2 Bursa Uludağ University, Faculty of Medicine, Department of Pulmonary Disease – Bursa, Turkey.; 3 Bursa Yuksek Ihtisas Training and Research Hospital, Department of Family Medicine – Bursa, Turkey.

**Keywords:** Academic performance, Cross-sectional study, Students, Internet addiction, Sleep quality, Smartphone addiction

## Abstract

**OBJECTIVE:**

In this study, we aimed to evaluate the relationships between Internet addiction, smartphone addiction, sleep quality, and academic success.

**METHODS:**

In this cross-sectional study, high-school students were surveyed to evaluate sleep quality, Internet addiction, and smartphone addiction. Students were queried about their demographics, and grade averages from the previous term were taken as an indicator of academic success.

**RESULTS:**

A total of 1,959 students were enrolled in this study, with 1,034 (52.8%) girls and 925 (47.2%) boys, and the median age of the participants was 16 (13–21) years. Multivariate analyses found that poor sleep quality in students who did not have breakfast before going to school was 1.58 times higher than those who did (p<0.001). Students who stayed in a dormitory had 1.79 times more poor sleep quality than those who stayed with their family, and a one-unit increase in the total score of the Young’s Internet Addiction Test short form resulted in a 1.08-fold increase (both, p<0.001).

**CONCLUSION:**

Our study has shown that students’ sleep quality was predicted to be lower if they stayed in a dormitory and skipped breakfast. In addition, Internet and smartphone addictions have a negative effect on sleep quality and academic performance.

## INTRODUCTION

Having entered people’s lives in the mid-1980s, the Internet gained rapid popularity and became a focus of attention among all age groups. In the past few years, there has been a remarkable expansion in the number of Internet users. Internet, which has become an important source of information, particularly for university students and adolescents, may lead to addiction in the said populations^1^. The Global Digital Report 2023 states that, of the world’s more than 8.01 billion people, 5.16 billion (64.4%) use the Internet and 5.44 billion (68%) use mobile phones^2^. According to the household information technologies usage survey results, it was determined that 95.5% of households in Turkey have access to the Internet from home in 2023^3^. In broad terms, Internet addiction can be characterized by the inability to overcome the urge toward excessive usage; the time spent offline losing its significance; irritability and aggressiveness in its absence; and the gradual impairment of one’s occupational, social, and family lives^1^, ^4^. School is a place of learning for adolescents. It not only plays an important role in education but also creates an environment for health promotion among young people.

Smartphones enhance daily lives by providing diverse social statuses and identities for younger people, compensating for shortcomings in social life. Adolescence is a critical period with biological, social, and psychological changes, leading to potential problems, making assessing smartphone addiction urgent. According to the Information Technologies Usage Research in Children 2021, 64.4% of children aged 6–15 years in Turkey used smartphones in 2021, with boys accounting for 65.7% and girls accounting for 63.0%^5^.

Sleep is a crucial daily activity affecting individuals’ quality of life and health, with physiological, psychological, and social dimensions. Adolescents need adequate sleep to regulate developmental functions and develop new skills, insights, and expectations. Sleep deprivation can increase daytime sleepiness and carelessness.

In this study, we aimed to evaluate the relationship between Internet and smartphone addictions, sleep quality, and academic success.

## METHODS

In line with the permission received from the Boyabat District Directorate of National Education and with the board of ethics approval given by the Clinical Research Ethics Board of Sinop University, a cross-sectional survey study was planned for February and March 2019. The survey comprised students from eight high schools in Boyabat, a rural region of Sinop province. Totally answering all questions in the surveys was regarded as satisfactorily completed.

### Participants

High-school students were surveyed to evaluate sleep quality, Internet addiction, and smartphone addiction. Students were queried about their demographics. Grade averages from the previous term were taken as an indicator of academic success. All of the questionnaires were filled out anonymously and on a voluntary basis, after the aim and scope of the study were explained to the students and were received from school administrations upon completion.

### Procedure

#### Pittsburgh Sleep Quality Index (PSQI)

The PSQI uses Likert and open-ended responses to assess sleep quality during the previous month. The PSQI measures subjective sleep quality, sleep latency, sleep length, habitual sleep efficiency, sleep disruptions, sleep medication, and daytime dysfunction. A total score of 0–21 is calculated by adding component values of 0–3. A global PSQI score over 5 indicates poor sleep and higher scores indicate poorer sleep quality. The reliability and validity of the Turkish version of PSQI were shown^6^.

#### Young’s Internet Addiction Test short form (YIAT-SF)

Developed by Young^1^ and shortened by Pawlikowski and friends^7^, YIAT-SF is a 5-point Likert (1=Never, 5=Very Frequent) type measure that consists of 12 questions. High scores on this measure show high levels of Internet addiction. The reliability and validity of the Turkish version were shown^8^.

#### Smart-phone Addiction Survey—Short Version (SAS-SV)

Consisting of 10 questions assessed by a 6-point Likert scale, SAS-SV was developed by Kwon et al., to measure the risk of smartphone addiction among adolescents^9^. Each item is rated on a scale ranging from 1 to 6. The overall score on the survey ranges from 10 to 60, with higher scores suggesting a greater risk of addiction. It is a single-factor survey without subscales. Cutoff scores are determined to be 31 for men and 33 for women. SAS-SV’s Turkish version was found to be reliable and valid^10^.

### Data analysis

The Shapiro-Wilk test was used to evaluate the normality of variable distribution. When continuous variables are normally distributed, they are expressed as mean and standard deviation. When they are not normally distributed, they are presented as median (min–max). Categorical variables were shown as n (%). According to normality test results, the Mann-Whitney U test was used for comparisons made between two groups and the Kruskal-Wallis test was used if more than two groups existed. To compare categorical variables, the Pearson chi-square test was performed. Correlation analysis was applied to investigate the relationships between continuous variables, and Spearman’s correlation coefficient was calculated. A backward stepwise conditional logistic regression was performed to determine independent predictors of students’ poor sleep quality. To measure model fit, Hosmer-Lemeshow goodness-of-fit statistics were used. The SPSS (IBM Corp. Released 2012. IBM SPSS Statistics for Windows, Version 21.0. Armonk, NY: IBM Corp.) software was used for statistical analysis, with p<0.05 taken as statistically significant.

## RESULTS

In accordance with the number of students enrolled at the high schools identified by the Boyabat District Directorate of National Education, 2,464 surveys were distributed to schools. A total of 1,959 students from eight schools in the district of Boyabat with 1,034 (52.8%) girls and 925 (47.2%) boys were enrolled in this study. Of the surveys distributed to schools, 79.5% were satisfactorily completed. The median age of the participants was 16 (13–21) years. The demographics of the students are presented in Table 1. Of the students, 9.08% (n=178) did not own smartphones.

**Table 1.** Socio-demographic characteristics of high-school students

Age (years)	n=1959
	16 [13–21]
Sex (girl)	1034 (52.8%)
**Year of study**	
Year 9	515 (26.3%)
Year 10	549 (28%)
Year 11	490 (25%)
Year 12	405 (20.7%)
**Mother’s level of education**	
Illiterate	66 (3.4%)
Primary	1182 (60.3%)
Secondary	419 (29.4%)
High school	227(11.6%)
University	65(3.3%)
**Father’s level of education**	
Illiterate	12 (0.6%)
Primary	823 (42%)
Secondary	508 (25.9%)
High school	61 (34.9%)
University	29 (16.6%)
**Mother’s occupation**	
Housewife	1643 (83.9%)
Worker	221 (11.3%)
Farmer	9 (0.5%)
Retired	8 (0.4%)
Tradesman	16 (0.8%)
Civil servant	62 (3.2%)
**Father’s occupation**	
Unemployed	64 (3.3%)
Worker	870 (44.4%)
Farmer	268 (13.7%)
Retired	123 (6.3%)
Tradesman	377 (19.3%)
Civil servant	257 (13.1%)
**Marital status of parents**	
Married	1849 (94.4%)
Separated	110 (5.6%)
**Income level**	
<2000	160 (8.2%)
2000–3000	780 (39.8%)
3000–4000	757 (38.6%)
>4000	261 (13.3%)
BMI (kg/m^2^)	21.05 ± 3.19
Breakfast (yes)	1311 (66.9%)
Own room (yes)	1230 (62.8%)
**Commute to school**	
By a vehicle	803 (41%)
Walking	1156 (59%)
**Resides**	
With family	1460 (74.5%)
In a dormitory	499 (25.5%)
**Previous term grade average**	72.8 ± 14.11
PSQI total score	6 [0–19]
YIAT-SF total score	26 [12–60]
SAS-SV total score	23 [10–60]

Continuous variables with normal distribution were presented as mean ± SD; non-normal variables were reported as median [min.: max.]. Categorical variables were shown as n (%). BMI: body mass index, PSQI: Pittsburgh Sleep Quality Index, YIAT-SF: Young’s Internet Addiction Test Short Form, SAS-SV: Smart-phone Addiction Survey—Short Version.

Assessment of factors related to grade averages showed that female students had higher grade averages than male students (78.0 [30.0–98.5] vs 70.0 [25.0–98.6], p<0.001); there was no difference between grade averages of students whose parents were together and of those whose parents were separated (74.0 [25.0–98.9] vs 72 [37–97], p<0.107); students who had breakfast in the mornings had higher averages than those who had not (75.0 [32.0–98.9] vs 72.0 [25.0–98.0], p<0.001); and students who had a room of their own had higher grade averages than those who had not (73.0 [35.0–98.0] vs 75.0 [25.0–98.9], p<0.001), while students who stayed in a dormitory had higher grade averages than students who stayed with their families (80.0 [35.0–98.9] vs 72.0 [25.0–98.5], p<0.001). Significant differences were found when the students’ grade averages were compared with their parents’ education levels, occupations, income levels, and study years (p<0.001 for each).

In terms of gender, no significant difference was found in YIAT-SF total scores (girls 26 [12–60]; boys 26 [12–55]; p<0.618). With the SAS-SV cutoff score taken to be 31 for men and 33 for women, 27.2% (n=281) of the girls, 28% (n=258) of the boys, and 27.5% (n=539) of all students were found to have smartphone addiction. In terms of smartphone addiction, there was no significant difference between the two sexes (p=0.685).

The end-of-term grade averages of the students were found to be positively correlated with age and BMI values (r=0.200, p<0.001 and r=0.050, p=0.029, respectively) and negatively correlated with PSQI, YIAT-SF, and SAS-SV total scores (r=0.062, p=0.006; r=0.083, p<0.001; and r=0.072, p<0.002, respectively). Assessing the relationship between quality of sleep and Internet and smartphone addiction, it was found that the PSQI total scores were positively correlated with the YIAT-SF and the SAS-SV total scores (r=0.362, p<0.001 and r=0.291, p<0.001, respectively). A positive correlation was found between the total scores of YIAT-SF and SAS-SV (r=0.707, p<0.001) (Table 2).

**Table 2.** Correlation analysis of the factors associated with grade point averages

Factor	r	p
Age, years	0.200	<0.001
BMI (kg/m^2^)	0.050	0.029
PSQI	-0.062	0.006
YIAT-SF total score	-0.083	<0.001
SAS-SV total score	-0.072	0.002

BMI: body mass index; PSQI: Pittsburgh Sleep Quality Index; YIAT-SF: Young’s Internet Addiction Test Short Form; SAS-SV: Smart-phone Addiction Survey—Short Version.

With a PSQI score of 5 or higher indicating poor sleep quality, it was found that 920 (47%) students had good and 1,039 (53%) students had bad sleep quality. Evaluating the factors that interact with poor sleep quality revealed that students with poor quality of sleep had lower grade averages (p=0.004) along with higher YIAT-SF total score and SAS-SV total score (p<0.001, for each) (Table 3).

**Table 3.** Factors related to sleep quality

	Sleep quality		p
	Poor (n=1039)	Good (n=920)	
Age (years)	16 [13-20]	16 [13-21]	<0.001^a^
Sex (girl)	563 (54.2%)	471 (51.2%)	0.189^c^
**Year of study**			<0.001^c^
Year 9	221 (21.3%)	294 (32%)	
Year 10	305 (29.4%)	224 (26.5%)	
Year 11	286 (27.5%)	204 (22.2%)	
Year 12	227 (21.8%)	178 (19.3%)	
**Marital status of parents**			0.472^c^
Married	977 (94%)	872 (94.8%)	
Separated	62 (6%)	48 (5.2%)	
BMI (kg/m^2^)	20.6 [14.5–36.2]	20.5 [14.4–36.2]	0.677^a^
Breakfast (yes)	636 (61.2%)	675 (73.4%)	<0.001^c^
Own room (yes)	654 (62.9%)	576 (62.6%)	0.888^c^
**Resides**			<0.001^c^
With family	740 (71.2%)	720 (78.3%)	
In a dormitory	299 (28.8%)	200 (21.7%)	
**Previous term grade average**	74 [25–98.9]	75 [32–98.5]	0.004^a^
YIAT-SF total score	29 [12–56]	23 [12–56]	<0.001^a^
SAS-SV total score	27 [10–60]	20 [10–58]	<0.001^a^

Continuous variables with non-normal variables were reported as median [min.: max.]. Categorical variables were shown as n (%). ^a^Mann-Whitney U test. ^c^Chi-square test. BMI: body mass index; YIAT-SF: Young’s Internet Addiction Test Short Form; SAS-SV: Smart-phone Addiction Survey—Short Version.

Multivariate analyses evaluated independent factors contributing to an increased risk of poor sleep quality. Poor sleep quality was 1.58 times higher in students who skipped breakfast (OR: 1.58, CI: 1.28–1.95, p<0.001). Being a year 10 student significantly increased poor sleep quality risk by 1.48-fold compared with being a year nine student (OR: 1.48, CI: 1.14–1.93, p=0.003), while year 11 students led to a 1.88-fold increase, and year 12 students led to a 1.98-fold increase (p<0.001 for each). Students who live in a dorm had 1.79 times the poor sleep quality of students who live with their families (OR: 1.79, CI: 1.42–2.26, p<0.001), and finally a one-unit increase in the total score of the YIAT-SF results in a 1.08-fold increase in poor sleep quality (OR: 1.08, CI: 1.06–1.09, p<0.001) (Table 4).

**Table 4.** Factors predicting poor sleep quality

	Univariate			Multivariate		
	OR	95%CI	p	OR	95%CI	p
Age, years	1.20	1.11–1.29	<0.001	–	–	–
Sex (girl)	1.12	0.94–1.35	0.186	–	–	–
**Year of study (Ref. year 9)**				–	–	–
Year 10	1.66	1.30–2.11	<0.001	1.48	1.14–1.93	0.003
Year 11	1.70	1.30–2.21	<0.001	1.88	1.43–2.48	<0.001
Year 12	1.86	1.45–2.39	<0.001	1.98	1.47–2.68	<0.001
Breakfast (Ref. yes)	1.75	1.44–2.11	<0.001	1.58	1.28–1.95	<0.001
Resides (Ref. with family)	1.45	1.18–1.78	<0.001	1.79	1.42–2.26	<0.001
Previous term grade average	0.99	0.98–0.99	<0.001	–	–	–
YIAT-SF total score	1.08	1.06–1.09	<0.001	1.08	1.06–1.09	<0.001
SAS-SV total score	1.04	1.03–1.05	<0.001	–	–	–

YIAT-SF: Young’s Internet Addiction Test Short Form; SAS-SV: Smart-phone Addiction Survey—Short Version.

## DISCUSSION

Aiming to evaluate the relationship between sleep quality and academic success to Internet and smartphone addiction among high-school students, our study found that the risk of poor sleep quality went up 1.6 times for students who did not have breakfast and 1.8 times for students who lived in a dormitory. Compared with year 9 students, year 10 students were found to increase the risk of poor sleep quality by 1.48 times, year 11 students by 1.88 times, and year 12 students by 1.98 times.

There was no difference between the sexes in terms of sleep quality. Arbinaga et al., have shown that poor sleep quality is more common by a factor of 1.52 among females^11^. In our study, it was determined that the average Internet and smartphone addiction survey scores of students with poor sleep quality were higher and their grade point averages were lower. Barahona et al., have found that, in general, students with poor quality of sleep perform poorly in academics^12^. In their study, Maheshwari et al., have shown 64.2% of participating students to be poor sleepers and that poor sleep negatively influenced academic performance^13^. Given that students in rural locations have limited access to the Internet and health information, guidance from health professionals can assist them in developing healthy habits^14^. When the relationship between quality of sleep and Internet and smartphone addictions was assessed, PSQI total scores were found to be positively correlated with YIAT-SF and SAS-SV total scores. In a study conducted by Chen YL et al., it was found that adolescents who had difficulties falling asleep or maintaining sleep were more susceptible to Internet addiction^15^. Our study has found a strong correlation between YIAT-SF and SAS-SV total scores. Similarly, in the study conducted by Tateno et al., a positive correlation was observed between YIAT-SF and SAS-SV^16^.

Significant differences in sleep quality and grade averages were observed between years of study. Female and senior-year students had higher grade averages. No difference was found between students with married or separated parents, as Brand et al., found no impact on children’s education^17^.

Our study has found that the students who have breakfast as opposed to those who do not and the students who have a room of their own as opposed to those who do not have higher grade averages. Meanwhile, in comparison with students staying with their own families, those students who stayed in a dormitory had higher grade averages. In a study by Araujo et al., the proximity of the dormitory to the school campus was suggested to have a positive impact on the academic success of the students^18^. While Soheilipour et al.^19^ found no significant relationship between having breakfast and academic success, Burns et al.^20^ have reported that the academic success of students who regularly had breakfast improved by a factor of 1.72.

Our study found that higher-income students perform better academically, with differences in grade averages influenced by parents’ education, occupations, and study year. Benner et al., have shown that children from families with higher socio-economic status perform better academic performance^21^. Similarly, Duan et al., have established that parents’ low socio-economic status has a negative impact on the child’s academic performance and socialization^22^. End-of-term grade averages of students were found to be positively correlated with their age and BMI while negatively correlated with PSQI, YIAT-SF, and SAS-SV total scores. A study of adolescents conducted in Korea has shown that students who perform well academically are less inclined toward Internet addiction^23^.

The study found no significant difference in smartphone addiction between male and female students. Students with addiction had higher YIAT-SF total scores and lower grade averages. Tateno et al., found smartphone addiction more prevalent among female students, with rates of 28% for girls and 22.8% for boys^16^. In another study, Kwon et al., have also shown smartphone addiction to be more prevalent among female students^9^.

In terms of the degree of Internet addiction, our study found no significant difference between the two sexes. In their study, Shek et al., found that Internet addiction negatively impacts academic performance^24^, while Haroon et al., found it more prevalent among girls^25^. Other studies on the subject have revealed male dominance in Internet addiction^26^, ^27^. Young et al., suggested that men use the Internet more frequently, which may explain the higher prevalence of Internet addiction among men^1^.

Our study has certain limitations. The participants consisted of Turkish adolescents; hence, the results cannot be generalized to other populations. As our study has made use of subjective tests, it might have led to response bias.

## CONCLUSION

Our study has shown that students’ sleep quality was predicted to be lower if they stayed in a dormitory and did not have breakfast. In addition, Internet and smartphone addictions had a negative effect on sleep quality and academic performance. In this respect, it is important to raise the awareness of parents and teachers who regularly interact with teenagers so that they can take the necessary steps to address increased Internet and smartphone usage during adolescence and the addiction that comes with it.
